# Fiscal federalism, inter-governmental transfers and public health– The case of Urban Local Bodies in India

**DOI:** 10.1016/j.ssmhs.2025.100123

**Published:** 2025-12

**Authors:** Samik Chowdhury

**Affiliations:** School of Public Policy and Governance, Dr. B. R. Ambedkar University, Delhi, India

**Keywords:** Fiscal federalism, Urban health, Inter-governmental transfers, Finance commission, Fifteenth finance commission, Urban local bodies, India, Public health, Primary health care

## Abstract

Public policy in India has historically ignored the potential of urban local bodies (ULBs), a constitutionally mandated third tier of Government, in addressing urban health issues. Constituted against the backdrop of an overwhelming pandemic, the Fifteenth Finance Commission (FFC) of India went beyond its conventional domain of recommending revenue sharing principles across different levels of Government, to make certain novel financial recommendations around decentralized provision of health services. This paper critically analyses the context, nature and scope of FFC recommendations for health to ULBs. In the process, the paper engages with diverse, yet connected themes like evolution of urban health policy in India, constitutional status and current fiscal situation of ULBs, their role in health service delivery, nature and composition of FFC grants for health and their status of implementation. A re-categorisation of FFC grants guided by a comprehensive understanding of health, shows that the grants are well designed to address the persistent, escalating as well as emerging public health problems of a rapidly urbanizing nation. Moreover, composition of these grants displays a clear emphasis on delivery of public health and primary health care services by local bodies - the tier of Government which is closest to its constituency. However, data from the first three years of the FFC period demonstrates inconsistencies in allocation, release and actual spending of health grants, which is likely to impact the realisation of its stated objectives.

## 1 Introduction

India has one of the most privatized health systems in the world (Government of India, 2023; Selvaraj et al., 2022; Hooda, 2020; Sengupta and Nundy, 2005). The private sector has not only established itself as a dominant provider of on-demand curative health care services, but also emerged as partner in the implementation of key national health programmes (Khan et al., 2024, Rao, 2024). Comprising of health care facilities of different size, scope and structure, location of these privately owned facilities is heavily skewed towards urban areas of the country. A fallout of this has been a near breakdown of public health and preventive, promotive and primary health care services, a constitutionally mandated function of the State (Constitution of India n.d.), particularly in the urban. Urban India is thus characterized on one hand by a dysfunctional referral chain even within the public health system and on the other, a diverse blend of privately owned tertiary health care providers ranging from informal clinics, formal clinics and nursing homes to corporate super-specialty hospitals, which have no pecuniary incentive to provide primary, preventive, and promotive health care (Mukhopadhyay et al., 2015, Baru, 1998). The heterogeneity of cost and quality of health care offered by this spectrum of health care providers, is reflected in persistent differences in health outcomes and treatment induced financial burden by spatial, economic and social attributes, across and even within urban settlements.Recent initiatives by the Union Government like the National Urban Health Mission (NUHM), and the Urban Health and Wellness Centre (HWC) component of the Ayushman Bharat^1^ scheme, aims to address the issue of insufficient primary health care services in the urban. Innovative approaches by some state governments viz. Mohalla Clinics (Delhi), Basti Dawakhana (Telangana), Sanjeevani Clinics (Madhya Pradesh), Namma clinics (Karnataka) etc. and community health initiatives steered by non-government organisations, have also contributed significantly to a growing interest in policies around urban health. Other than the provision of primary health care closer to the community, most of these initiatives reveal a crucial acknowledgment of a rapidly changing disease profile of the urban population and their evolving health care needs.

A potentially momentous institutional contribution to this “movement” has come from the Fifteenth Finance Commission (FFC). The Finance Commissions are appointed by the President of India every five years, whose primary function is to recommend the sharing of tax proceeds and grants between the three tiers of Government – Centre, State and Local, to address vertical and horizontal fiscal imbalances and inequities in the levels of public services across jurisdictions. The FFC, formed against the backdrop of the Covid pandemic however marked a break from the previous Finance Commissions and recommended a significantly large specific purpose grant for health to the local Governments in India – rural and urban, to be spent over five years (2021–26), for plugging the critical gaps in the health system at the primary health care level. In addition to the specific grants for health, a considerable share of the routine grants to the local bodies have been conditionally linked to select public health goals (Government of India, 2020, Government of India, 2021).

While the novel health specific grants for local bodies have received attention in popular media (Lahariya, 2021, Sikarwar, 2021) and to a limited extent academia (Chathukulam and Joseph, 2022), the conditional grants linked to crucial public health goals, have largely gone unnoticed. This has resulted in an underappreciation of the push towards decentralized provision of basic health services by an institution (Finance Commission), whose terms of reference did not necessarily require it to do so. Chairman of the FFC has on record listed inadequacy of public outlays for health, lack of prioritization of health in state budgets, low share of primary health care in health budgets and inter-State variations in health spending and outcomes as the key challenges facing India’s health sector (The Statesman, 2022). These pertinent apprehensions coupled with the horrifying experience of the pandemic, may have been the driving force behind the FFC recommendations on decentralised provision of health services. The FFC’s local body grants for health may also be interpreted as a recognition of the failure of the higher tiers of the Government to ensure public health and primary health care to its populace, particularly in times of emergency.

Against this backdrop, this paper critically analyses the context, nature and scope of FFC grants for health to urban local bodies in India. While doing so, the paper engages with the evolution of urban health policy in India (3.1), constitutional status and current fiscal situation of urban local bodies in India, their competence and capacity to provide the proposed health services (3.2 & 3.3), role of the Finance Commission in local government finances (3.4), contribution of urban local bodies (ULBs) in health service delivery (3.5), the nature and composition of FFC grants for health, the status of their implementation and possible challenges in utilisation of the same (3.6, 3.7, 3.8, 3.9). A potentially significant contribution of this paper is a re-estimation of FFC grants for health, based on a more expansive understanding of health, that goes beyond mere health care. The subsequent sections of the paper elaborate on each of these aspects, starting with a description of the materials and methods used to build up the arguments.

## 2 Materials and methods

The paper is essentially descriptive, but analytical. A major constraint we faced was the lack of adequate, disaggregated and reliable data and information on local governments, particularly their financial aspects. The paper therefore engages with diverse materials and data sources to build and support its arguments. These include the Constitution of India for normative frameworks, Finance Commission reports and their background papers for financial devolution to local bodies over time, the Reserve Bank of India (RBI) report on finances of the local bodies for their revenue and expenditure profiles, policy documents from the health sector for a review of policies specific to urban health, answers to parliamentary questions for current status of fund utilization, Press Information Bureau (PIB) releases for scheme related information, background papers of the National Health Accounts (NHA) for health financing by ULBs and technical and operational guidelines for implementation of FFC grants for the standard operating procedures.

The paper also makes a conceptual departure from the generic understanding (and even of the FFC) of health services comprising solely of health care. Essential public health functions like water, sanitation, air quality, solid waste management etc. have been integrated with health care for a comprehensive definition of health services. The methodological implication of this is that the FFC grants for health had to be re-classified, and their shares recalculated, to have an accurate account of inter-governmental transfers for health services by the FFC.

## 3 Results

### 3.1 Urban health in India - a review of key policy pronouncements

Urban health had a steady presence in policy, since India’s independence, but not always to its advantage. Till about the 5th Five Year Plan^2^ (1974–78), policy makers were concerned that the meagre health facilities in the country displayed a marked urban bias, and that urban health services could have been expanding at the cost of the rural, which was where the majority of population resided. The Sixth Plan (1980–85), that came against the backdrop of the momentous Alma Ata Declaration (1978) of ‘health for all’ with a focus on primary, preventive and promotive health services, tried to reign in the growth of curative services in the urban areas (Kumar et al., 2016). However, private sector presence in curative health care continued to increase incrementally with a corresponding withdrawal of the State from provision of the same. This led to an increase in the cost of health care and a huge unmet demand for primary health care particularly among the urban poor, many of whom were the aspirational migrants. Health inequity within the city increased steadily (Sharma et al., 2020, Speizer et al., 2012, Chowdhury, 2011).

The reversal in policy outlook towards urban health, in terms of the recognition of the need for basic urban health services, came as late as in 1982 with the Krishnan Committee report. The report recommended the establishment of a health post with a Doctor, a Public Health Nurse, four Auxiliary Nurse Midwives, four Multi-Purpose Workers, and 25 Community Health Workers, for an urban population of 50,000 and called it the Urban Revamping Scheme (Kapadia-Kundu and Kanitkar, 2002).

A decade later in 1993, the World Bank supported India Population Project (IPP) VIII focused on reducing infant and maternal deaths in urban slums by expanding the service delivery system to the slum population through the construction of additional facilities. The IPP-VIII created 479 Urban Health Posts, 85 Maternity Homes and 244 Sub Centers in Mumbai, Chennai, Delhi, Bengaluru, Hyderabad and Kolkata (Government of India, 2013). These facilities contributed significantly to urban health infrastructure in these rapidly growing cities, and received continued support from subsequent government programmes on urban health as well (Aggarwal et al., 2008).

The Ninth Plan (1997–2002) also emphasized urban healthcare, especially the absence of primary healthcare and the complete reliance on higher levels of services, even for minor ailments. It showed that contrary to popular perception, health indicators in urban slums were often worse compared to even rural and tribal areas of India. It proposed to address this issue through provision of primary healthcare services, especially in slums and providing referral linkages at higher levels (Duggal, 2002).

The National Health Policy (NHP) of 2002 acknowledged the inadequacies and inefficiencies in public health services in urban areas and its implications for out of pocket (OOP) health expenses among poor households. NHP 2002 also identified multisectoral linkages between drinking water, sanitation, air pollution etc. with the changing disease burden in urban settings. In terms of physical infrastructure, the policy recommended setting up of a two-tiered urban primary healthcare system following population norms and establishment of a referral system. (Nundy, 2005)

The National Urban Health Mission (NUHM) launched in May 2013 as a sub-mission under the National Health Mission brought a renewed focus on the health needs of urban populations, particularly the marginalized sections, in view of the rapidly privatizing health care space and its consequences on health care access and household finances. It aimed to provide comprehensive primary healthcare services in urban areas, through Urban Primary Health Centers (U-PHCs), Urban Community Health Centers (U-CHCs) (which can act as first referral units), strong outreach services and accessible frontline health workers in all State capitals, district headquarters and cities/towns with a population of more than 50000, thus largely aiming to replicate the model that had been operating in rural India since 2005 (Government of India, 2013).

The next major policy push for urban health came from the National Health Policy (NHP), 2017. With a dedicated section on urban health, NHP 2017 proposed to move away from token interventions to guaranteed primary health care delivery and referral support with special focus on poor populations living in listed and unlisted slums, other vulnerable populations such as homeless, rag-pickers, street children, rickshaw pullers, construction workers, sex workers and temporary migrants. The policy advocated scaling up of National Urban Health Mission (NUHM) to cover the entire urban population within the next five years with sustained financing. It also proposed convergence among the social and environmental determinants of health like air pollution, solid waste management, water quality, occupational safety, road safety, housing, vector control, and reduction of violence and urban stress (MOHFW^3^-Government of India, 2017).

Finally, in 2018, the Government of India's announced the Ayushman Bharat programme, one of its components being the creation of 1,50,000 Health and Wellness Centres (HWCs) by upgrading the pre-existing Sub Centres and Primary Health Centres in rural and urban areas. These centres would deliver Comprehensive Primary Health Care (CPHC) responsive to the changing disease burden of the country, bring healthcare closer to people, and include free essential drugs and diagnostic services. In urban areas, the pre-existing health facilities such as Urban Primary Health Centres (UPHCs) and/or dispensaries were to be converted to Health & Wellness Centres, or new facilities were to be set up (Government of India, 2019).

In all this, a role for the Government that was closest to its constituency – the urban local bodies (ULBs), was conspicuous by its absence. The argument in favour of decentralization for better public service delivery is rooted in the principle of subsidiarity which essentially states that service is best provided by a Government that is in closest contact with the beneficiary (Rao and Bird, 2010, Kersbergen and Verbeek, 2004). It is likely to have better information about people and their service requirements, more flexibility, higher efficiency and cost-effectiveness, improved accountability, and can promote healthy competition across adjacent jurisdictions. On the other hand, decentralization could also lead to issues like deviation from national political and economic objectives, loss of operational economies of scale, inability to internalise externalities, political and economic instability, inequitable service levels across jurisdictions, and elite capture (Pius Kulipossa, 2004). Most importantly, effective service provision requires an optimum combination of funds, functions, and functionaries which is not necessarily guaranteed by political decentralization alone.

Several countries have experimented with decentralization of public health and health care functions with different models and varying degrees of success. Brazil’s Sistema Único de Saúde (SUS) or Unified Health System, established in 1989 is managed entirely by the state and local governments. Indonesia’s health system underwent significant changes in 2001, when managerial and financial responsibilities of primary health care provision shifted from the federal government to the districts. South Africa’s approach to decentralization since early nineties, revolved around local clinics and hospitals for the delivery of health care and implementation of the National Health Insurance (NHI) scheme (Winchester and King, 2018) by district health authorities. In UK, health system decentralization meant each constituent country manages its own National Health Service (NHS), public health and health care delivery systems and were accountable to their respective parliaments. The impact of decentralization on health financing and outcomes in these countries have been mixed, due to a host of systemic issues like inconsistencies in political commitment, funding inadequacy, inequity in resource allocation, lack of monitoring and evaluation, capacity constraints, fragmentation of services, staff shortages, inefficient procurement policies, political influence and corruption (Pimentel et al., 2023, Jurberg, 2008, Atkinson and Haran, 2004, Brennan and Abimbola, 2023, Kristiansen and Santoso, 2006, Winchester and King, 2018, McIntyre and Klugman, 2003, Sparer et al., 2011).

### 3.2 Fiscal decentralization and ULBs in India

ULB’s in India comprise of municipal corporations for larger urban areas, municipalities for smaller urban areas, and nagar panchayats/town panchayats/notified area council for areas in transition from rural to urban. ULBs, along with rural local bodies (RLB), have historically been accepted as democratic units of self-governance in India. However, it was only after the 73rd and 74th Constitutional Amendment Act of 1992 that they were formally recognized as the third tier of Government, after the Union and the State governments. Currently, there are over four thousand ULBs in India (Table 1), which are very diverse in their demographic characteristics. For instance, population density in municipal corporations is four times that of nagar panchayats and twice that of municipalities.

**Table 1:** Urban local bodies in India, 2019.

	Number of ULB’s	Population (millions)	Population density (population per sq kms)
All Urban	4259	358*	
Municipal Corporations	209	172	7514
Municipal Councils	2171	112	3189
Nagar Panchayats	1879	32	1681

Source: Ahluwalia et al#, (2019)

* Total urban population equals the population residing in these statutory ULB’s plus those residing in Census towns (areas without ULB but defined as urban as per Census criteria) and special urban administrative units e.g., Cantonment Boards (civic administration body in India under control of the Ministry of Defence).

The 74th Constitutional Amendment Act also listed a set of 18 functions (Table 2) under the 12th Schedule that were to be assigned to the ULBs by the State governments. States were free to devolve selected functions from the Schedule to the ULBs while keeping the rest to themselves. This resulted in differences across states in levels of functional decentralization.

**Table 2:** Functions Listed in the 12th Schedule of the Constitution.

1. Urban planning including town planning
2. Regulation of land-use and construction of buildings
3. Planning for economic and social development
4. Roads and bridges
5. Water supply - domestic, industrial and commercial
6. Public health, sanitation, conservancy and solid waste management
7. Fire services
8. Urban forestry, protection of environment and ecology
9. Safeguarding the interests of weaker sections of the society including the handicapped
10. Slum improvement and upgradation
11. Urban poverty alleviation
12. Provision of urban amenities and facilities - parks, gardens and playgrounds
13. Promotion of cultural, educational and aesthetic aspects
14. Burials and burial grounds, cremations, cremation grounds and electric crematoriums
15. Cattle pounds, prevention of cruelty to animals
16. Vital statistics including registration of births and deaths
17. Public amenities including street lighting, parking lots, bus stops and public conven
18. Regulation of slaughterhouses and tanneries

The Amendment did very little in terms of fiscal decentralization, as these functions were not matched by any comparable assignment of revenue sources to the ULBs. Although the Constitution has some provisions for augmentation of resources of the ULBs by their respective state governments, actual effective transfer of revenue sources under these provisions has been limited (RBI,^4^ Reserve Bank of India, 2022). This was probably the key reason why despite their locational advantage, ULBs never assumed a leadership role in delivery of health services.

### 3.3 Fiscal profile of ULB’s

Municipal revenues/expenditures in India amount to just around one per cent of GDP (Table 3), much lower than comparable countries e.g., municipal revenues/expenditures account for 7.4 per cent of GDP in Brazil and 6 per cent of GDP in South Africa (Reserve Bank of India, 2022). Own revenues account for just 43 % of the total revenues of ULB which essentially implies fiscal dependence on higher tiers of Government, given the limited scope of municipal borrowing. Revenue from property tax is 15 % of total revenues, 35 % of total own revenues and 60 % of total tax revenues of ULBs. Thus, property tax which is collected by most ULBs, is the single most important source of revenue over which the ULBs have an apparent control. This is particularly so after the introduction of the Goods and Services Tax (GST) that subsumed few buoyant local taxes e.g. entertainment tax, with no corresponding compensation to ULB’s (Das Gupta, 2020). Transfers to the ULBs are made by both the Union and the State governments, with State transfers accounting for almost a third of their total revenues. Close to two-thirds of Union Government transfers to ULBs are guided by the Central Finance Commission recommendations, the remaining being through different centrally sponsored schemes.^5^

**Table 3:** Fiscal profile of ULBs (All).

**Indicators (units)**	**All ULB's (2017–18)**
Total Revenue (Rs Crore)	171697
Per capita Total Revenue (Rs)	4624
Total Revenue as share of GDP (%)	1.0
Own Revenue as share of Total Revenue (%)	42.7
Transfers as share of Total revenue (%)	44.3
Market Borrowings as share of Total revenue (%)	2.2
Other Revenue as share of Total revenue (%)	10.7
Tax Revenue as share of Total Revenue (%)	25.0
Property tax as share of Total Revenue (%)	14.9
Non-tax Revenue as share of Total Revenue (%)	17.7
Central Transfers as share of Total Revenue (%)	12.0
CFC grants as share of Total Central Transfers (%)	59.9
Other Central Transfers as share of Total Central Transfers (%)	40.1
State Transfers as share of Total Revenue (%)	32.4
Total Expenditure (Rs Crore^a^)	132553
Per capita Total Expenditure (Rs)	3570
Total Expenditure as share of GDP (%)	0.8
Revenue Expenditure as share of Total Expenditure (%)	59.0
Capital Expenditure as share of Total Expenditure (%)	41.0

a 1 crore = 10 million

Revenue expenditure, generally comprising salaries, establishment, operation and maintenance has a larger share of total expenditure than capital expenditure which usually creates new assets and is considered as an indicator of quality of public expenditure.

In addition to the low levels, municipal finances in India display considerable variations across size class of cities/towns. The average per capita revenue and expenditure of the larger municipal corporations is more than twice that of the smaller nagar panchayats (Table 4).

**Table 4:** Key fiscal indicators by ULB type.

	All ULB's	Municipal Corporations	Municipal Councils	Nagar Panchayats
Total Revenue (Rs Crore)	171697	116726	44926	10046
Per capita Total Revenue (Rs)	4624	5783	3421	2635
Total Expenditure (Rs Crore)	132553	91559	33214	7779
Per capita Total Expenditure (Rs)	3570	4536	2529	2040

**Fig. 1:**
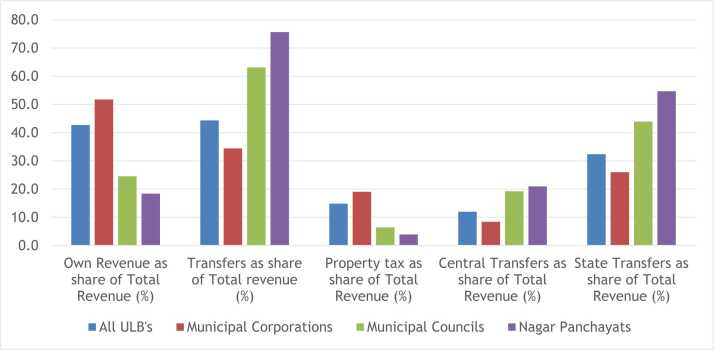
Key fiscal indicators by ULB type.

Source: Same as Table 3.

The share of own revenue in total revenue, commonly considered as a measure of fiscal autonomy, also display a distinct gradient (Fig. 1). For smaller ULBs, almost three fourth of their revenue comes from transfers from the higher tiers of government. The smaller the ULB type, the higher the share of both Central and State transfers.

The poor financial capacity and autonomy of ULBs have implications for the level, quality and efficiency of public service delivery. Moreover, larger urban centres are characterized by overlapping jurisdictions of different tiers of Government and semi-autonomous parastatal bodies, which adversely impacts quality of services and blur accountability (Gandhi and Pethe, 2017) pathways. As a result, while cities have been the drivers of economic development in India over the last three decades, with substantial migratory pull, level and quality of essential public services failed to keep pace with the resultant surge in demand.

The mismatch between expenditure responsibilities and revenue sources of ULBs was acknowledged early and this led to the incorporation of “*measures to augment the Consolidated Fund of a State to supplement the resources of its municipalities*” as one of the terms of references (TOR) for the Finance Commissions, a constitutional body appointed every five years by the President of India to recommend the design and quantum of intergovernmental fiscal transfers.

### 3.4 Role of Finance Commissions

The Indian Constitution provides for the setting up of a Finance Commission on the orders of the President of India, to make recommendation on the distribution of net proceeds of taxes between the Union and the States, allocation between the States of respective shares of such proceeds; grants- in-aid to the States and measures needed to supplement the resources of the rural and urban local bodies during the five-yearly award period (Constitution of India n.d.).

**Fig. 2:**
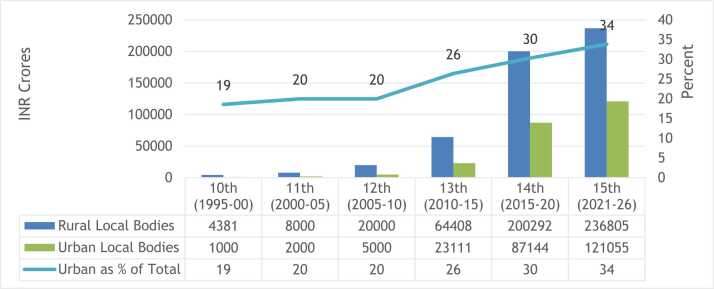
Grants recommeded for Local Bodies by Finance Commissions.

Source: Author, using data from reports of respective Finance Commissions

The urban local bodies were made part of the TOR for FCs only after the 74th Constitutional Amendment, 1993 and therefore specific grants for ULBs were recommended 10th FC (1995–2000) onwards. Fig. 2 shows that the share of ULBs in grants recommended for all local bodies – rural and urban, has been increasing over successive FC’s. The most recent FC has recommended more than a third of total devolution to ULBs in alignment with the changing demographic characteristics of the country.

### 3.5 ULB’s and health service delivery – status and prospects

The 12th schedule of the constitution enlists public health, sanitation, conservancy and solid waste management as functions that may be devolved to the ULBs (Table 2). However, this does not include health care. Therefore primary, secondary and in some instances even tertiary health care facilities that are provided by some ULBs, has a direct association with their size, fiscal capacity and the extent of functional autonomy they enjoy. In other words, on one hand larger and financially solvent ULBs like Mumbai, Bengaluru, Chennai, Bhubaneshwar, Kolkata, Ahmedabad etc. run a graded health care system that comprises of urban health posts, dispensaries, specialist clinics, general and even multispecialty hospitals, while on the other, health services of smaller ULBs are restricted, often inadequately, to vector control and other public health functions like drinking water, sanitation and solid waste management (Janagraha, 2023). The reasons, other than functional autonomy, are mainly low financial capacity of ULBs in poorer states, shortage of quality human resources and lack of flexibility and capacity for health planning at a decentralized level.

**Fig. 3:**
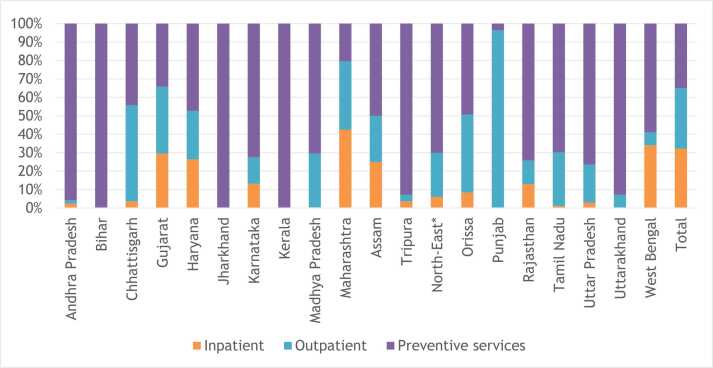
Composition of health expenditure by ULBs in selected states.

Source: Author, using data from the ULB report, National Health Accounts

In terms of health care spending, data from the NHA of India show that ULBs spent INR 12,630 crores on health in 2021–22, which amounted to only about 1.6 % of the total current health expenditure^6^ of the country. However, this share has steadily increased from 0.9 % in 2013–14. Very little evidence exists on the composition of health spending by ULBs other than municipal corporations, due to the lack of relevant financial data in the public domain. The municipal budgets or financial statements are often aggregative and lacks a uniform structure which renders analysis of health spending at this level, almost impossible. The National Health Accounts cell which is entrusted with the task of estimating total health expenditure in the country, attempted to capture health spending by local Governments by sending questionnaires to a sample of ULBs in the country. The ULB report of the National Health Accounts showed that ULBs spend only about 4 percent of their total expenditure on health and except for a few states, services provided were mostly preventive in nature (Fig. 3). ULBs of only a few states had their own health facilities, that provided comprehensive health care. Also, there exists substantial differences in health expenditure by ULBs across states – while Maharashtra spends close to Rs. 500 per capita, Jharkhand spends less than Rs. 10 (PHFI, 2016).

### 3.6 The Fifteenth Finance Commission and the Health Sector

While some of the previous Finance Commissions have also recommended specific purpose fiscal transfers for the health sector, these were directed towards the state Governments (Fan et al., 2018) and the quantum of grants were not significantly large or commensurate to the then status of health outcomes in the country. Constituted against the backdrop of the devastating pandemic, the FFC (2021–26) was the first Finance Commission to explicitly recognize the importance of health and the need to involve and empower local governments to deal with issues ranging from public health interventions to delivery of health care. The recognition was also backed by resources and the range of functional and financial delegation was on full display for ULBs. Given the limited role played by ULBs so far, in provision of health services in the country, the FFC health grants has a lot of potential and therefore deserve a comprehensive analysis of their status and prospects.

As has been mentioned in the introduction, while the health specific grants for local bodies have received popular attention, conditional grants linked to other crucial public health goals, have largely gone unnoticed. This paper therefore applies a health lens to the total quantum of FFC grants, instead of restrictively looking at just the health specific grants. We find that the FFC allocations for health (Fig. 3) can be recategorized broadly into four components – (1) primary healthcare-specific grants to local governments, (2) grants linked to specific public health goals for local governments (3) secondary/tertiary level health sector grants to States and (4) specific purpose grants for health to States based on approved projects pertaining to the health sector.

The primary healthcare-specific grants to local governments have six components - two of which are specific to the ULBs and the remaining four to RLBs. These six components broadly cover delivery of primary health care, in alignment with the Union government's vision of comprehensive primary health care under Ayushman Bharat (Lahariya, 2020). The public health grants to local governments are directed towards issues like sanitation, drinking water, solid waste management, rainwater harvesting, water recycling and ambient air quality. The health sector grants to State governments are tilted towards secondary and tertiary care and medical education with components like critical care hospitals, district integrated public health labs, DNB courses in district hospitals and training of allied health care workforce. Finally, the specific purpose health grants to States are for implementation of already approved project proposals e.g., upgradation of Patna Medical College in Bihar, setting up of nursing institute in Manipur etc.

In absolute terms, FFC recommended a sum of Rs. 4,36,361 crores to local governments, rural and urban, for the period 2021–26 out of which, as per our estimates, Rs. 3,00,047 or close to 69 % has been recommended for either public health and allied sectors or primary health care infrastructure. Out of this, Rs. 1,14,035 crores, i.e., 38 % has been awarded to the ULBs. In addition, the FFC has recommended Rs. 31755 crores to state governments for specific secondary and tertiary health infrastructure as mentioned earlier. Thus, the total amount that has been recommended for health by the FFC for the period 2021–26, comes to Rs. 3,31,802 crores (Rs. 66,360 crores per annum), which is 32 % of total grants^7^ recommended by the FFC. Given that the total health expenditure in India in the year 2019–20 was Rs. 6,55,822 crores, 41.4 % of it being public expenditure, the share of FFC grants for health (public health and health care) in total health expenditure and total Government health expenditure amounts to 10 % and 24 %, respectively.

**Fig. 4:**
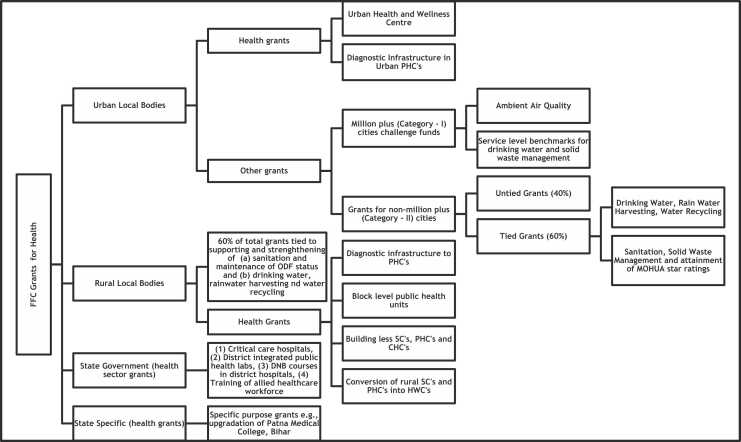
FFC grants for health (Source: Author, using FFC recommendations).

The FFC grants for health may also be dissected using two analytical lenses - the levels of health services and the extent of decentralization. Assuming public health, primary, secondary and tertiary healthcare to be the different levels of health services respectively, we find that the FFC has recommended 68 % percent of its health grants for public health, 21 % for primary health care, 10 % on secondary/tertiary health care and the remaining 1 % on health specific projects by selected states (Fig. 4).

On applying the decentralization lens, it is seen that the local government share of total health grants comes to 90 % (ULB:34 %, RLB:56 %). Together, this marks a clear prioritization of health in inter-governmental fiscal flows and an unprecedented emphasis towards fiscal decentralization for the provision of public health and primary health care functions.

### 3.7 FFC health grants to ULB’s: An elaboration

As discussed earlier, the FFC grants to ULBs can be classified into health and non-health grants. The health grants can be further classified into public health and primary health infrastructure. The FFC makes a distinction between million plus cities and the rest, in its recommendations concerning public health aspects. For million plus cities, recommendations have been made in the form of a Million-plus Cities Challenge Fund (Rs. 38,196 crores). Almost one-third of this fund is for realizing ambient air quality based on recognized parameters, while the remaining two-thirds is for meeting service level benchmarks on drinking water supply, sanitation, solid waste management and water conservation. Cities with less than a million population were to receive Rs. 82,859 crores, 60 percent of which was tied to public health priorities as in the case of million-plus cities, while the remaining 40 percent were untied grants which can be used by the ULBs for the functions listed in the 12th schedule (Table 2), except for the salary component.; (Table 5)

**Table 5:** FFC grants to ULBs.

**Components**	**Absolute amount (Rs. Crores)**	**Percentage composition (%)**
1. **Health grants**	114035	77.5
a. **Public health (i + ii)**	87912	59.7
i. Million plus cities (1 +2)	38196	26.0
1. Ambient air quality	12139	8.2
2. Attainment of service level benchmarks on drinking water, sanitation, solid waste management and water conservation	26057	17.7
i. Others (non-million plus) cities (3 + 4)	49716	33.8
3. Sanitation, solid waste management and attainment of service level benchmarks	24858	16.9
4. Drinking water, water conservation	24858	16.9
b. **Primary health care infrastructure (iii + iv)**	26123	17.7
i. Support for diagnostic infrastructure to Urban PHCs	2095	1.4
i. Urban health and wellness centres	24028	16.3
2. **Non-health grants (Untied grants for non-million plus cities)**	33143	22.5
**Total FFC grants to ULBs (A + B)**	**147178**	**100.0**

The FFC grants for primary health care infrastructure to ULBs has two components. Major part of it is for setting up urban HWCs and polyclinics for decentralized delivery of primary health care to marginalized and uncovered areas within the cities. A minor share goes to setting up diagnostic infrastructure in urban PHC’s, as a significant step towards comprehensive primary health care, a national priority as per the Ayushman Bharat programme. It is important to note that to avail all grants except those for primary health care infrastructure, the ULBs are required to comply with certain conditionalities viz. availability of audited accounts, notification of floor rates of property tax and growth of property tax revenue commensurate with the growth in state domestic product.

### 3.8 Operationalisation of FFC health sector grants

The ULB’s have been entrusted with operationalization of the health grant. However, since small and medium ULB’s have been mostly performing rudimentary public health functions, it was felt that they would need some hand holding for the components pertaining to primary health infrastructure. Therefore, technical and operational guidelines (TOG) have been issued by MOHFW that lays down the overarching principles for planning and implementation of FFC health grants through local government (Government of India, 2021).

Though the funds are to be spent by the local governments, the TOG has delineated specific roles to be played by the ULB, the district administration, the state urban development department, the state health department and their union government counterparts and the union finance ministry. A district level committee (DLC) comprising officials from the urban affairs and health department including the chief medical officer of the district will prepare a district health action plan (DHAP) based on local level plans received from the ULB’s. Similarly in each state, a state level committee (SLC) headed by the Chief Secretary with representation from the health and urban affairs departments of the state government, will prepare a State level plan based on the DHAPs. The state level plans will be finally submitted to a national level committee (NLC) comprising of Secretary, MOHFW and Principal Secretaries of Health of all states. The NLC is the approval granting authority for the state level plans received from the SLC’s. It also draws a time line of deliverables and outcomes for each of the five years along with a mechanism for fund flow and utilisation of these grants and issues necessary technical guidance to the states from time to time. Decision on procurement of medical equipments and items for diagnostic services are to be made by the SLC, which may also opt for decentralized procurement with approval from the NLC, conditional on economies of scale, efficiency, and adherence to standards and procedure. A ULB may utilize the health sector grant in convergence with other Centrally Sponsored Schemes (CSS) or top it up with their own resources. The objective however would be to enhance coverage of services within a ULB jurisdiction or improvement of quality of services, and not duplication of existing schemes. Also, the health sector grant should not be used as a State contribution towards a CSS. The state wise annual resource envelope for each component over the next five years have already been specified by the FFC. The funds released under the FFC grants for each Financial Year have to be utilized in the same year.

### 3.9 Status of FFC health sector grants

Half way into the grant period (2021–26), this is probably an appropriate time to assess the flow of FFC funds to the health sector through local governments. A major obstacle to this is the lack of data at the local level. The data limitation prevents us from evaluating actual spending, outputs and outcome at the local level. We however perform a preliminary analysis of the FFC health grant releases made by the Finance Ministry to each state government, for subsequent sharing with their local governments –rural and urban, for the two-year period between 2021‐22 and 2023‐24. This is based on data available from an answer to one of the parliamentary questions. Table 6 contains the results of this analysis.

**Table 6:** Status of FFC grants to local bodies (ULBs and RLBs) for health – all states aggregate.

	**2021–22**	**2022–23**	**2021–22 to 2022–23**
Grants recommended by the FFC (Rs. Crore)	13192	13192	26384
Grants approved by the NLC (Rs. Crore)	13154	12980	26134
Grants released by the DoE, MoF (Rs. Crore)	12252	3309	15561
Approved as share of recommended (%)	99.7	98.4	99.1
Release as share of approved (%)	93.1	25.5	59.5
Release as share of recommended (%)	92.9	25.1	59.0

In order to understand the contents of Table 6, it may be useful to recall that the total FFC recommended grants for health^8^ through local bodies is Rs. 70,051 crores^9^ over the five-year period 2021–26, which has been broken down to annual allocations for each state and specified in the FFC report. The annual allocations form the resource envelope for states for preparing an annual consolidated health plan by assimilating the plans of their respective local bodies. The consolidated plan of each state is brought to the NLC for approval. After NLC approval, the Department of Expenditure (DoE), Ministry of Finance (MoF) is directed to release the approved amounts to the states who then transfers the same to the local bodies as per their submitted (and approved) local health plans.

**Fig. 5:**
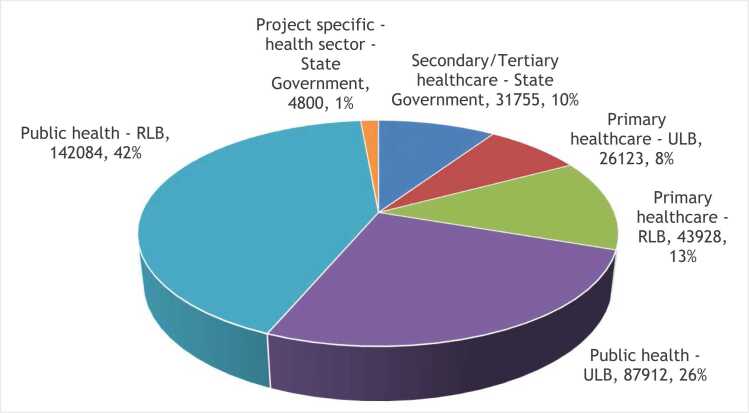
Health grants recommended by the XVth Finance Commission - A Reclassification.

Source: Author’s calculation using data from FFC report

Table 6 shows that in the first two years of the FFC, almost the entire recommended grant amount was approved by the NLC, which indicates good coordination between different levels of Government and the designated committees, given the complex process around these approvals. It also shows that the felt need for a health system upgrade, albeit through selected channels (see footnote 11), was quite high. Such high levels of felt need, readiness and competence may be attributed to the pandemic experience of the immediate previous year, which inarguably offered a reality check for public health systems all across the country. However, when we look at actual release the results are not as encouraging. In the first two years of the FFC only 60 percent of NLC approved health grants were released to states for their respective local governments. The share in fact declines to 25 % in 2022–23 meaning that out of the total approved grants, only a quarter was released by the MoF. There also exists huge inter-state variation in the share (%) of release in total recommendation (Fig. 5). Fifteen out of the twenty-one major states have so far received just half of their total approved grants for the period 2021–23, or less. Tamil Nadu, Telangana and West Bengal seem to be the only states with 100 % of its approved grants released by the Ministry of Finance. On the other extreme is Maharashtra, a large state, which has so far received just 20 percent of its approved amount. The state level variations in approvals and release are an outcome of a complex interplay of political-economic factors which include administrative capacity, degree and quality of communication and coordination across levels of government, financial management practices, technological infrastructure, political alignment with the Union Government, compliance with conditionalities for fund transfers etc. A deeper investigation is required to find the relative strength of each of these factors, which is beyond the scope of the current study.

**Fig. 6:**
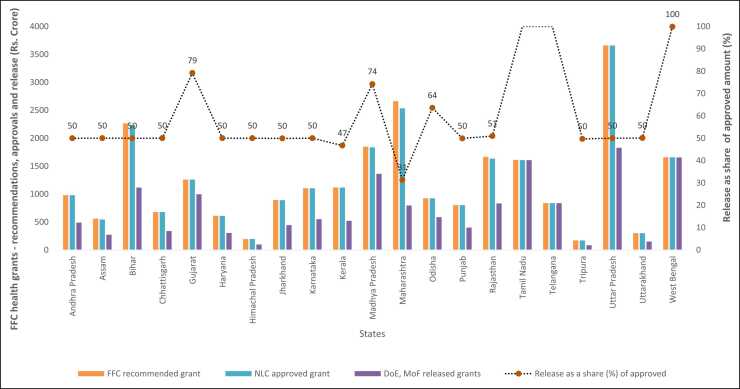
FFC health grants to local bodies - recommendations, approvals and release, 2021–22–2022–23.

Source: Author, using data from FFC report (Government of India, 2020) and Rajya Sabha Unstarred Question No. 277 (Government of India, 2023)

The only study we found, that looked at utilisation of FFC health grants within a state, found a shocking underutilisation of health grants in Kerala, the model state for decentralized healthcare. The reasons for underutilization, identified by this study on the basis of key informant interviews, ranged from political tensions between levels of Government, delay in disbursement of grants, competition among corporators regarding location of new urban primary health centres, corruption in recruitments and purchases, unfamiliarity with the TOG, inadequate scope to incorporate local needs and so on (Chathukulam et al., 2024); (Fig. 6)

## 4 Discussion and conclusion

Urban health has faced historical neglect in India. Better aggregate health outcomes vis-à-vis the rural, and massive presence of the private sector of different hues in the curative health care segment, led to an under-recognition of the widespread inequities in access to health care, quality of health services, health outcomes and financial burden of health care in the urban. This was accompanied by a simultaneous withdrawal of the State from the realm of urban health leading to a vacuum in essential primary, preventive, promotive and public health services. Recent policy pronouncements acknowledge urban health as a distinct problem area and incorporates the urban, explicitly in its agenda. However, these pronouncements are largely silent on the role that the constitutionally mandated third tier of Government i.e., the ULBs can potentially play. Even the flagship programmes on the health sector had well defined roles for the union government, state government and even autonomous bodies and parastatals while giving the democratically elected third tier a convenient miss.

It is in this context that the recommendations of the FFC are significant. Coming against the backdrop of a devastating pandemic, the FFC, whose primary terms of reference is design of revenue sharing principles across different levels of Government, made some novel recommendations pertaining to the health sector, which were largely aligned to the current health policy landscape of the country and backed by financial allocations.

On a careful analysis of the FFC recommendations, we find that Rs. 3,31,802 crores (Rs. 66,360 crores per annum) have been allocated for public health and health care services, for the period 2021–26. This amounts to 32 % of all grants recommended by FFC, 10 % of the total health expenditure and 24 % of the total Government health expenditure in the country. On applying the decentralization lens, we see that the local government share of total health grants comes to 90 % (ULB:34 %, RLB:56 %). In terms of levels of health services, it is found that the FFC has recommended 68 % percent of its health grants for public health, 21 % for primary health care, 10 % on secondary/tertiary health care and the remaining 1 % on health specific projects by selected states, which could not be neatly classified into one of the three levels.

From the perspective of decentralization and urban health, we find that the FFC recommended a sum of Rs. 4,36,361 crores to local governments, rural and urban, for the period 2021–26 out of which, as per our estimates, Rs. 3,00,047 crores, or close to 69 % has been recommended for health. Out of this, Rs. 1,14,035 crores, i.e., 38 % has been awarded to the ULBs. This also constitutes 78 % of overall grants recommended for ULBs by the FFC. Although grants for primary health care facilities are novel and therefore popular, the major share (60 %) of ULB grants are actually directed towards public health interventions like water, sanitation, solid waste management and ambient air quality which cover the persistent, escalating as well as emerging problems of a rapidly urbanizing country.

Together, this marks a distinct prioritization of health in inter-governmental fiscal flows and an unprecedented emphasis towards fiscal decentralization for the provision of public health and primary health care services. This significant reorientation in delivery of public health and primary health care services in a mixed disease burden scenario like urban India, has largely gone unnoticed in popular media and academia, possibly because the thrust does not come from the MOHFW, but from a very unlikely source, the Finance Commission.

However, roughly halfway into the grant period, we find that only 60 percent of NLC approved health grants were released to states for their respective local governments. In the year 2022–23, only a quarter of the total approved health grants for local governments, was released by the MoF to states. Moreover, substantial inter-state variation exists in release as a share of recommendation. Fifteen out of the twenty-one major states have so far received just half of their total approved grants for the period 2021–23, or less.

Unavailability of municipal level data, disaggregated by functional classification of expenditure, is a huge constraint, that studies on municipal finance in India generally face. This study is not an exception either. Therefore, the study had to be restricted to analysis of aggregate municipal finance data at the state level. Even details about FFC recommendations, approvals and release of health grants were available up to the state level. This prevented us from exploring intra-state disparities in ULB finances in general and for health in particular, which could be significant, based on the size of the state, its governance performance and absorptive capacity of its ULBs. Even though all municipalities in the country go through a budgeting process, there is no consistency in terms of accounting practices, layout of the budget document, level of disaggregation of revenue and expenditure line items or availability of this information in public domain. Given that a stated purpose of FFC grants is to ensure comparable levels of public services across the country, absence of granular data on municipal finances and health outcomes would make a rigorous evaluation of FFC recommendations and their aftermath, a very difficult task. Future work in this area may take up thoughtfully sampled case studies of selected municipalities across the country, to better understand issues like flow of FFC funds, prioritization of health needs, utilization of financial transfers and impacts on health outcome.

The FFC health grants for ULBs (and RLBs) are a potential gamechanger as far as the idea of decentralized provision of health is concerned. However, it needs to be seen in the context of the absorptive capacity of these local bodies, given the historical indifference they have faced in allocation of funds, functions and functionaries for carrying out their constitutionally mandated responsibilities. Few key issues need to be discussed in this respect.

One, health grants to ULBs, other than the specific primary health care infrastructure grants, have been made conditional on broadly two aspects – own revenue generation and presentation of reliable financial information in the public domain. Enhancement of own revenue through fixation and regular updation of property values and tax rates and alignment of the same with general macroeconomic conditions of a state can go a long way in empowering ULBs. This is not the first time that such conditionalities have been imposed for release of Central grants to ULBs and therefore it remains to be seen how the ULBs respond to it given that majority of the grants are to be used for public health outcomes, which are not as remunerative electorally, as, say, physical infrastructure.

Enhancement of own revenues, which has been set as a broad conditionality for availing some of these grants, will also have implications for sustainability of primary health care services of the ULBs which will be funded through the FFC grants only for a period of five years, till 2026. The TOG issued by MOHFW says that a ULB may utilize the health sector grant in convergence with other Centrally Sponsored Schemes (CSS) or top it up with their own resources.

Availability of reliable financial information is also an area where ULBs were found wanting. This has prevented researchers and policy makers from conducting any meaningful analysis and evaluation of their performance. Putting that as a conditionality for receiving funds is smart policy, although this has been tried in case of centrally sponsored schemes like JNNURM in the past, with limited success.

Two, the FFC has provided the total resource envelope for ULBs of each state in its report; the inter-se distribution of these grants (public health, primary health care and non-health) across ULBs, have been left to the state governments. This distribution is of crucial importance as ULBs within a state are very different in terms of their size and capacity to handle financial resources and functional responsibilities. This will have implications for equity in the level and quality of health services delivered by ULBs within a state.

An associated factor is that of capacity of the functionaries. It may be too much to expect a small municipality with no experience of health care service delivery, to prepare health action plans, operationalise HWC’s, arrange diagnostic facilities, make necessary procurements, deal with human resource issues and other generic issues of operation and maintenance. Considerable amount of hand holding in the form of training etc. may be needed in the initial period which needs to be provided by the higher levels of Government. The state governments should grab this opportunity to empower smaller ULBs for delivery of essential health services rather than the larger ones which are financially self-reliant and usually have an established health care system in place. Besides, the FFC grant may not make a substantial difference to the revenue profiles of larger ULBs.

Three, even though the FFC makes all the right noises in terms of decentralized delivery of public health and primary health care services, the technical and operational guidelines to operationalize this grant, issued by the MOHFW evokes a tendency towards centralization. The entire process of ULB level health action plans moving up through multiple administrative layers viz. the DLC, the SLC and the NLC involving state departments and central ministries like health, urban affairs and finance is likely to introduce unnecessary complexities and delays in fund transfer and betray the spirit of the FFC recommendations. Some of that is already evident from our analysis of release of these grants. While this may be justified in the name of hand holding, monitoring and accountability, the state governments can explore the possibility of capacity building of a more sustainable nature at the local level that could cut down the time involved in review of plans at multiple levels of governance.

Finally, FC grants are mandated transfers from the Union to local governments to address vertical and horizontal fiscal imbalances and ensure comparable level of services across jurisdictions. While it is exciting to see that a dominant share of these transfers this time has been linked to health, this should not replace the Central share of existing centrally sponsored schemes in the health sector with similar mandates.

Under the popular Ayushman Bharat programme, primary health care facilities are expected to reorient themselves by incorporating prevention and management of NCDs, mental health etc., under a comprehensive primary health care model, going beyond their historical role of a provider of services related to maternal and child health and communicable diseases. The explicit FFC grants for primary health care infrastructure to local bodies is expected to facilitate this reorientation. But, possibly an under-appreciated but more critical component of FFC health grants are those, that are directed towards longstanding public health issues like water, sanitation, solid waste management and ambient air quality, which are likely to escalate as India urbanizes rapidly in the coming decades.

## CRediT authorship contribution statement

**Samik Chowdhury:** Writing – original draft, Supervision, Project administration, Formal analysis, Visualization, Software, Methodology, Data curation, Writing – review & editing, Validation, Resources, Investigation, Conceptualization.

## Declaration of Generative AI and AI-assisted technologies in the writing process

The author declares that no generative AI or AI-assisted technologies were used in the writing process.

## Declaration of Competing Interest

The author declares no financial and personal relationships with other people or organizations that could inappropriately influence (bias) this work.

The author declares that the work has not been published previously and is not under consideration for publication elsewhere. If accepted, it will not be published elsewhere in the same form, in English or in any other language, including electronically without the written consent of the copyright-holder.

## Footnotes

1 Started in 2018, the Ayushman Bharat programme has two components (1) comprehensive primary health care through Health and Wellness Centres that will eventually replace the existing public primary health care system which apparently has a narrow focus in terms of changing disease burden, and (2) Pradhan Mantri Jan Arogya Yojana (PMJAY) or the prime minister’s public health insurance scheme, which aims to provide health insurance coverage for secondary and tertiary health care to the vulnerable populaton defined in terms of economic status, age and selected occupational characteristics.

2 Since its independence in 1947 till the year 2017, India had five-year plans for social and economic development which were formulated, executed and monitored by the Planning Commission.

3 MOHFW is the abbreviation for Ministry of Health and Family Welfare

4 RBI is an abbreviation for the Reserve Bank of India

5 Centrally sponsored schemes (CSS), designed to address specific development goals of the country, are formulated by the Union Government but implemented by the State Governments or their agencies. Funding of CSS’s is done by the Union and the States in the ratio 60:40 for general states and 75:25 for some special category states.

6 NHA defines current health expenditures (CHE) as recurrent expenditures for healthcare purposes. Thus CHE is total health expenditures (THE) net of all capital expenditures.

7 FFC grants comprise of (1) Post-devolution revenue deficit grants (2) Disaster relief grants (3) Grants to local governments (4) State specific grants and (5) Sector specific grants. Out of these, all except (3) are directed towards state governments.

8 This amount is what the FFC report states as the total health grants to local bodies. The constituents of this grant have been elaborated in footnote 11. This amount is not based on the more holistic definition of health that we have adopted in this study, that includes public health aspects like water, sanitation, solid waste management, ambient air quality etc.

9 The total FFC health grant for local bodies is to be spent on six distinct components of health infrastructure – four for RLBs and two for ULBs. The rural components are (1) support for diagnostic infrastructure to primary health care facilities (2) establishing block level public health units, (3) grants for building-less primary health care facilities and (4) conversion of rural sub health centres and primary health centres into health & wellness centres (HWCs). The urban components are (1) support for diagnostic infrastructure to urban primary health care facilities and (2) grants for urban health and wellness centres (UHWCs).
